# The effect of age, sex and obesity on fundamental motor skills among 4 to 6 years-old children

**DOI:** 10.12669/pjms.292.3069

**Published:** 2013-04

**Authors:** Roshanak Vameghi, Amir Shams, Parvane Shamsipour Dehkordi

**Affiliations:** 1 Roshanak Vameghi, Pediatric Neurorehabilitation Research Center, University of Social Welfare and Rehabilitation Sciences, Tehran, Iran.; 2 Amir Shams, Department of Physical Education and Sport Sciences, Shahid Beheshti University, Tehran, Iran.; 3 Parvane Shamsipour Dehkordi, Department of Physical Education and Sport Sciences, Shahid Beheshti University, Tehran, Iran.

**Keywords:** Obesity, Overweight, FMS, OSU-SIGMA

## Abstract

***Objective:*** To examine the effect of age, sex and obesity on Fundamental Motor Skills (FMS) in 4 to 6 years-old children.

***Methodology:*** A total of 400 preschool children (200 boys and 200 girls) between the ages of 4 to 6 years old participated in this research. Subjects were selected through multi-stage cluster random sampling. Fundamental motor skills (FMS) were assessed with using the OSU-SIGMA scale. Body mass index (BMI) was directly measured from height(m)^2^/weight(kg) for each child and based on CDC growth charts, normal weight, overweight and obesity were defined.

***Results:*** The results showed that age and sex variables were a significant effect on walking and running skills, but BMI was not significant (P>0.05). Also, these variables had a significant effect on jumping, skipping, hopping and ladder climbing. In both ages, boys in jumping and ladder climbing skills were better than girls, but the girls were better in skipping and hopping skills (P<0.05). Moreover, the results showed that age and BMI variables have a significant effect on stair climbing skill, but sex was not significant (P>0.05). For object control skills, the results showed that age and sex variables were a significant effect on catching and throwing skills, but BMI was not significant (P>0.05). Finally, the age, sex and BMI variables were a significant effect on kicking and sticking skills.

***Conclusion:*** This research demonstrated that boys performed better than girls, and both overweight and obese children have lower performance than normal children.

## INTRODUCTION

The prevalence of overweight and obesity among children is rapidly increasing worldwide.^1^^,^^2^ The prevalence of children obesity in 2010 was estimated about 46 percent in the Americas and 38 percent in European regions. This increase is alarming, because obesity associated with health disease (e.g. hypertension, cardiovascular disease, type 2 diabetes, high blood pressure, abnormal lipid profiles) and psychological risks.^3^^-^^5^ Studies have shown that adult obesity is directly associated with childhood obesity so that in obese children, the prevalence of adult obesity is 2 to 3 times more than normal children.^6^

Several studies have found that obesity is a multi-factorial disease and several factors (e.g. genetics, poor nutrition, psychological and environmental factors) are involved in its development. Moreover, these studies have mentioned that the lack of adequate physical activity is one of the important reasons for obesity.^7^^,^^8^ Cools et al also believe that we are often unaware on importance of physical activity, but games and physical activity are vital and inseparable part of human life. So that is critical and crucial for physical, cognitive, social and motor development.^9^^,^^10^ Thus, the main elements of motor development, in addition to its biological foundation, are fundamental motor skills (FMS) that involved the gross and fine motor skills.^11^^,^^12^

 Researchers believed that preschool years (ages 3-6 years) are critical period to a development and proficiency of FMS.^11^^,^^13^ The acquisition of these skills are developmentally sequenced and are dependent in several internal and external factors (including biological, social, environmental, psychological, cognitive, etc.).^13^ Furthermore, researchers believed that the progress in these skills depends on the range of play or game experiences and organized programs.^13^ Failure to develop and improvement of FMS during the preschool and elementary school years often leads to privation and failure the master skills during adulthood.^14^ It’s implies that the poor performance in FMS may jeopardize future participation in sport and physical activity. This problem also leads to decreased movement and mobility in children and increase the prevalence of overweight and obesity risks.^10^^,^^15^^,^^16^ Accordingly, several studies reported that the obesity is negatively related to the performance of FMS, so that the obese children have delays in this skills.^7^^,^^17^

Researchers believe that in addition to overweight and obesity, age and sex variables are important impact on the development of FMS.^18^ As such in several studies, researchers found that with increasing age, children in catching and kicking skills were more matured, but in these studies no significant difference has been reported between sex.^18^^,^^19^ Moreover, Butterfield and Loovis stated that the performance of FMS in boys were significantly better than girls.^20^^,^^21^ Other studies have also found that in all ages, girls are consistently better than boys in hopping and skipping skills.^22^^,^^23^ In most studies done in this area, researchers have separately examined the effect of age, sex, and obesity factors on FMS. Some studies that have considered all of these factors, have only evaluated one or two FMS. As such the comprehensive research conducted in this area for evaluation of these factors seems to be necessary. Therefore, the purpose of this study was to examine the effect of age, sex and obesity on FMS in 4 to 6 years-old children.

## METHODOLOGY

A total of 400 preschool children (200 boys and 200 girls) between the ages of 4 to 6 years participated in this research. Subjects were selected through multi-stage cluster random sampling from five geographic regions of Tehran (north, south, west, east and center). With children dressed in light clothing, barefoot, stood erect against a wall and feet flat on the floor, standing height was measured to the nearest 0.1 cm using a portable stadiometer and body mass was measured to the nearest 0.1 kg using a digital scale (Seca Model, Germany). From those two measurements, BMI was calculated as weight (kg)/height (m)^2^ for each subject and converted in to the BMI Z-scores and percentiles for age and sex based on Centers for Disease Control and Prevention, 2000, (CDC) growth charts. Normal weight, overweight and obesity were defined by 5^th^≤BMI≥85^th^ percentile, 85^th^≤BMI≥95^th^ percentile and BMI≥95^th^ percentile, respectively.^24^

To measure FMS, each child was individually administered the Ohio State University Scale of Intra Gross Motor Assessment (OSU-SIGMA). The OSU-SIGMA is a criterion-referenced assessment scale and designed to assess eleven FMS in age range of 2.5 to 14 years old. This FMS divided to locomotor skills (walking, running, jumping, hopping, skipping, stair climbing and ladder climbing) and objective control skills (throwing, catching striking and kicking) and presented in four developmental levels.^18^^,^^19^ The study was conducted according to the Ethical Committee of the University of Social Welfare and Rehabilitation Sciences.

The data have been analyzed with using descriptive (mean and percentages) and inferential statistics such as multiple regression test^18-21^ at the significance level of P<0.05.

## RESULTS

The results related to performance levels of FMS by age and sex are presented in Tables I and II. The results of the locomotor skills are presented in Table-III. Accordingly, the results of multiple regression showed that age (5-4 and 6-5 years) and sex (boys and girls) variables were a significant effect on walking and running skills, but BMI was not significant (P>0.05). In addition, the boys performed better than girls in both age groups. The results also showed that age, sex and BMI variables had a significant effect on jumping, skipping, hopping and ladder climbing. In both ages, boys in jumping and ladder climbing skills were better than girls, but the girls were better in skipping and hopping skills. Moreover, overweight and obese children, in both ages, have lower performance than normal children. Finally, the results showed that age and BMI variables had a significant effect on stair climbing skill, but sex was not significant (P>0.05). 

**Table-I:** The levels of FMS in 4-5 years-old children

	*Girls*				*Boys*			
*Performance Levels*								
FMS	1	2	3	4	1	2	3	4
Walking	----	----	55%	45%	----	----	31%	69%
Running	----	----	73%	27%	----	----	55%	45%
Jumping	----	28%	72%	----	----	14%	82%	4%
Hopping	1%	75%	24%	----	62%	37%	1%	----
Skipping	77%	23%	----	----	83%	17%	----	----
Stair Climbing	----	----	57%	43%	----	----	57%	43%
Ladder Climbing	2%	64%	34%	----	2%	51%	47%	----
Catching	47%	49%	4%	----	25%	62%	13%	----
Throwing	47%	53%	----	----	36%	62%	2%	----
Striking	91%	9%	----	----	86%	14%	----	----
Kicking	62%	38%	----	----	47%	53%	----	----

**Table-II:** The levels of FMS in 5-6 years-old children

	*Girls*				*Boys*			
*Performance Levels*								
**FMS**	**1**	**2**	**3**	**4**	**1**	**2**	**3**	**4**
Walking	----	----	35%	65%	----	----	19%	81%
Running	----	----	49%	51%	----	----	38%	61%
Jumping	----	9%	82%	9%	----	65%	35%	----
Hopping	5%	41%	54%	----	22%	48%	30%	----
Skipping	5%	51%	38%	6%	33%	58%	9%	----
Stair Climbing	----	----	42%	58%	----	----	37%	63%
Ladder Climbing	----	63%	37%	----	----	40%	52%	8%
Catching	14%	62%	24%	----	5%	43%	52%	----
Throwing	29%	51%	20%	----	1%	30%	69%	----
Striking	53%	40%	7%	----	5%	48%	48%	----
Kicking	26%	58%	16%	----	6%	54%	40%	----

**Table-III:** Multiple regression analysis for FMS by age, sex and BMI in 4 to 6 years-old children

				*Independent variables*		
*Dependent variables*	*R* ^2^	*F*	*df*	*Age*	*Sex*	*BMI*
Walking	0.08	11.126	396	----	----	----
B	----	----	----	0.126*	0.202*	-0.070
Beta	----	----	----	0.170*	0.201*	-0.075
Running	0.065	9.082	396	----	----	----
B	----	----	----	0.204*	0.144*	0.030
Beta	----	----	----	0.205*	0.145*	0.031
Jumping	0.215	36.059	396	----	----	----
B	----	----	----	0.367*	0.268*	-0.093*
Beta	----	----	----	0.370*	0.270*	-0.095*
Hopping	0.327	64.102	396	----	----	----
B	----	----	----	0.480*	-0.630*	-0.146*
Beta	----	----	----	0.341*	-0.447*	-0.106*
Skipping	0.404	89.415	396	----	----	----
B	----	----	----	0.880*	0.300*	-0.144*
Beta	----	----	----	0.596*	0.203*	-0.099*
Stair Climbing	0.070	9.989	396	----	----	----
B	----	----	----	0.133*	0.083	-0.216*
Beta	----	----	----	0.132*	0.082	-0.212*
Ladder Climbing	0.073	10.467	396	----	----	----
B	----	----	----	0.145*	0.225*	-0.147*
Beta	----	----	----	0.131*	0.203*	-0.135*
Catching	0.234	40.232	396	----	----	----
B	----	----	----	0.559*	0.339*	0.024
Beta	----	----	----	0.412*	0.250*	0.018
Throwing	0.346	69.684	396	----	----	----
B	----	----	----	0.697*	0.447*	0.089
Beta	----	----	----	0.489*	0.314*	0.064
Striking	0.490	126.761	396	----	----	----
B	----	----	----	0.874*	-0.473*	0.219*
Beta	----	----	----	0.606*	-0.328*	0.156*
Kicking	0.341	68.300	396	----	----	----
B	----	----	----	0.675*	0.305*	-0.279*
Beta	----	----	----	0.504*	0.228*	-0.213*

*Significant at the level of P<0.05

The results of object control skills are presented in Table-III. It showed that age and sex variables had a significant effect on catching and throwing skills, but BMI was not significant (P>0.05). In these skills, boys were better than girls. On the other hand, the results showed that age, sex and BMI variables were a significant effect on kicking and striking skills. Boys are better performance than girls. Overweight and obese children had lower performance than normal children.

## DISCUSSION

The results of present study related to locomotor skills are consistent with finding of Catenassi et al. These researchers reported no significant difference in this area, perhaps because the children in walking and running skills are practice and mastery faster than the other FMS.^25^ Gabbard mentioned that these skills are less affected by BMI, because the proficiency in this skills leads to better transport of excess fat in overweight and obesity in children.^12^ Also, D’Hondt et al. found that the performance of motor skills are more difficult, and more components are weaker in obese children. Therefore, the walking and running skills are fewer motor components, thus, are less affected by BMI.^2^

The results related to the jumping, skipping, hopping and ladder climbing skills are consistent with the results of Morrison et al.^7^ In another study, Southall et al found that BMI was an adverse effect on FMS, especially on jumping skill.^11^ Accordingly, in the proper performance of these skills, BMI and body mass transport have a great impact, so that overweight and obese children have a lower performance. On the other hand, due to these skills are more difficult and more components have negative effect on BMI. Moreover, in hopping and skipping skills, the results showed that girls have better performance than boys, but only 6% of girls 5-6 years were preformed skipping skill at the level 4 (mature level). These findings are consistent with results of Woodard and Surburg who stated that the six years-old children could not perform these skills at the mature level.^23^

Okely et al stated that the performance of FMS that requires more motor components are difficult for overweight and obese children.^5^ It has been shown that overweight and obese children find it more difficult to move their limb or larger body mass against gravity. In addition, overweight and obese children are more likely to have orthopedic changes such as flat feet, which may lead to greater pain when performed the FMS or plays.^11^

For object control skills, the results of this research are consistent with the findings of Butterfield and coauthors.^18^^-^^21^ In this context, researchers reported that BMI has limited the range of motion in arms so that excess fat can also limit the movement in the shoulder and leads to poor performance in overweight and obese children.^2^ In general, the results of this study revealed that age, sex and BMI had a significant effect on the performance of FMS. These results are usually explained and described from a mechanical point of view. Accordingly, the body fat affects on body geometry and increases the mass of different body segments. Hence, noncontributory mass could lead to biomechanical movement inefficiency and could be detrimental for motor proficiency,^2^ that is what our results indicate. On the other hand, the negative impact of BMI on fundamental motor skills can be explained by some other mechanisms. Overweight and obese children are often failure to perform the difficult activities and FMS. Thus, this leads to a decreased regular physical activity and plays. These children are less likely to be physically active and show preference for sedentary pastime.
